# The Pretherapy Endothelial Activation and Stress Index (EASIX) in Patients with Newly Diagnosed Acute Myeloid Leukemia: A Pilot Study

**DOI:** 10.3390/diagnostics16193117

**Published:** 2026-09-25

**Authors:** Øystein Bruserud, Guido Smits, Silje Johansen, Espen Talseth Skar, Øystein Wendelbo, Håkon Reikvam

**Affiliations:** 1 Department of Clinical Science, University of Bergen, P.O. Box 7804, N-5020 Bergen, Norway; silje.johansen@haraldsplass.no (S.J.); espen.talseth.skar@gmail.com (E.T.S.); 2 Department of Medicine, Haukeland University Hospital, N-5021 Bergen, Norway; oystein.wendelbo@helse-bergen.no; 3 K.G. Jebsen Center for Myeloid Blood Cancer, Department of Clinical Science, University of Bergen, P.O. Box 7804, N-5021 Bergen, Norway; gusmi0020@uib.no; 4 Department of Medicine, Haraldsplass Deaconess Hospital, P.O. Box 6165, N-5892 Bergen, Norway; 5 Department of Nursing, VID Specialized University, Bergen Campus, Ulriksdal 10, N-5009 Bergen, Norway

**Keywords:** acute myeloid leukemia, chemotherapy, allogeneic stem cell transplantation, EASIX, endothelial activation and stress index

## Abstract

**Background/Objectives**: The endothelial activation and stress index (EASIX; serum creatinine times lactate dehydrogenase/peripheral blood platelet count) seems to reflect endothelial cell activation/dysfunction. Furthermore, endothelial cells support acute myeloid leukemia (AML) cell proliferation and survival, and high bone marrow microvascular density is associated with adverse prognosis in human AML. **Methods**: We investigated the pretreatment EASIX score in 160 AML patients (promyelocytic variants excluded) at the time of diagnosis before initiation of intensive cytarabine-based anti-AML therapy. **Results**: We detected a significant association between the pretreatment EASIX score and the monocytic AML cell phenotype (Fisher’s test, *p* = 0.0007). A weaker association was observed between a high pretreatment EASIX score and C-reactive protein levels (Spearman’s test, *p* = 0.017). We could not detect any significant difference in 5-year overall survival when comparing patient subsets with a pretreatment EASIX score below versus above the median cohort level (Kaplan–Meier’s test, *p* = 0.84), and the overall survival did not differ when comparing overall survival for the two contrasting upper and lower quartiles either (*p* = 0.88). Furthermore, the 5-year posttransplant survival did not differ between allotransplant recipients (*n* = 58) with a pretreatment (i.e., at the time of first diagnosis) EASIX score below versus above the cohort median level (*p* = 0.55). Finally, the 5-year overall survival did not differ when comparing patients with low versus high modified EASIX scores either (*p* = 0.46). **Conclusions**: Our study suggests that the pretherapy EASIX score does not have any prognostic impact in non-promyelocytic AML patients receiving conventional intensive AML therapy. This is different from patients with promyelocytic AML variants, in whom a high EASIX score is associated with increased mortality. However, EASIX scoring should be further investigated in AML patients who receive new targeted therapies, including BCL2-inhibition, which is less effective in monocytic AML variants.

## 1. Introduction

The endothelial activation and stress index (EASIX; lactate dehydrogenase (LD) × creatinine/peripheral blood platelet count) is an indirect marker of endothelial activation/dysfunction/damage [1,2]. A high pretransplant score is associated with decreased survival mainly due to increased non-relapse mortality for patients receiving allogeneic stem cell transplantation (for an updated review, see [1]). The EASIX score has a prognostic impact even in nonmalignant diseases, including infections and cardiovascular diseases [3,4,5]. Furthermore, EASIX scoring at the time of first diagnosis can also be associated with prognosis in certain solid tumors [6] and even in certain hematological malignancies, including myelodysplastic syndromes (MDS) [7], lymphoma [8], and the acute promyelocytic leukemia (APL) variant of acute myeloid leukemia (AML) [9], when tested at first diagnosis or before cellular therapies other than allotransplantation. Angiogenesis is regarded as a part of carcinogenesis/leukemogenesis, and these malignancies are thus characterized by cancer-associated local angiogenesis and, at the same time, a prognostic impact of the EASIX score. Similarly, AML is also characterized both by leukemic cell proliferation/survival that is supported by the bidirectional crosstalk between leukemic and endothelial cells [10,11,12], by increased microvessel density in the bone marrow microenvironment and by an adverse prognostic impact of this local angiogenic response for AML patients receiving intensive and potentially curative treatment [13,14,15,16,17].

Cancer-associated angiogenesis is a part of the leukemogenesis in AML [10]. The aim of the present study was to investigate whether the pretherapy EASIX score at the time of first AML diagnosis (APL excluded) has a prognostic impact in patients receiving potentially curative intensive antileukemic therapy. However, unlike certain other hematological malignancies (including the APL variant of AML), we did not detect any significant association between pretherapy EASIX levels and survival in non-APL variants of human AML. A possible hypothesis for this difference could be that (i) molecular mechanisms involved in the communication between cancer and endothelial cells vary between malignancies and for certain malignancies may not be reflected in the EASIX score, and/or that (ii) the EASIX score may also be influenced by masking factors without prognostic impact.

## 2. Materials and Methods

### 2.1. Identification and Inclusion of Patients

The Regional Ethics Committee (REK Vest) classified this study as quality assurance (REK-Vest number 404696); written consent was therefore not considered necessary. The hospital data protection officer at Haukeland University Hospital also approved the study, and all identifiable patient information was stored on a secure hospital server.

Haukeland University Hospital is responsible for diagnosis and treatment of AML for all patients in a defined geographical area. This retrospective single-center study is based on 160 consecutive patients receiving potentially curative intensive antileukemic therapy during the period 2004–2021, with all patients thus having a follow-up period of at least four years. Patients with APL (AML-M3) were excluded from the study. Using a digital search tool based on diagnostic codes stored in the electronic patient journal system, we extracted every patient with a registered AML diagnosis during the period 2001 to 2021. Patients under the age of 18 years at the time of diagnosis and patients not receiving cytarabine plus anthracycline-based induction therapy were excluded from the study. We initially obtained 1300 subjects with a diagnosis of AML. After removing duplicates, patients where laboratory serum creatinine/serum LD/peripheral blood platelet count were not attainable before the start of induction treatment, patients with AML diagnosis or treatment sat in a different center, and patients with APL, we were left with 160 patients.

### 2.2. Calculation of the EASIX Score

The EASIX score was calculated by the formula lactate dehydrogenase (LD) serum level (IU/L) × creatinine serum level (µmol/L)/peripheral blood platelet count (10^9^/L) and thereafter log2-transformed (Tables S1 and S2). Both calculated and log2-transformed values are presented throughout the article. The modified EASIX score (mEASIX) was calculated by replacing the creatinine level with the serum C-reactive protein level (CRP, mg/L).

### 2.3. Statistical Analyses

The Mann–Whitney U test was used to compare continuous parameters, Fisher’s exact test (for two groups) or Chi-Square test (more than two groups) for comparisons of categorized data and Spearman’s rank tests for correlation analyses.

Survival was measured in months from the defining event (i.e., AML diagnosis or stem cell transplantation) to either time of death or the end of the 5-year observation period. Patients who died within 30 days were given a survival time of 1 month; for patients surviving ≥1 month, survival was measured as completed months. Overall survival was truncated at 60 months (i.e., 5 years) and analyzed using the Kaplan–Meier method. Differences between survival curves were assessed using the log-rank test, and these statistical analyses were performed in R (v4.5.0).

Statistical significance was defined as *p* < 0.05.

## 3. Results

### 3.1. Patient Characteristics

Our study included 160 patients; their characteristics are summarized in Table 1 and Table S1. They represent all adult (i.e., above 18 years of age) AML patients from a defined geographical area with an available EASIX score who received intensive and potentially curative anti-AML therapy during a defined time period (December 2004–September 2021). Patients received induction chemotherapy including an anthracycline in combination with cytarabine; the consolidation therapy was either high-dose cytarabine alone (3 g/m^2^) or combinatory chemotherapy including intermediate dose cytarabine (1 g/m^2^) [18,19].

We regard the frequencies of males/females, karyotype/*FLT3*/*NPM1* abnormalities, as well as frequencies of patients with primary resistant disease, early death and long-term overall survival to be as expected compared with other AML studies [18,19,20,21].

### 3.2. The Variation Range of the Three Individual EASIX Parameters

The pretreatment EASIX score showed a wide variation between patients, with a median absolute calculated value of 495 and a range of 17–11,061 (log2-transformed values: median 9.0, range 4.1–13.4). Furthermore, the pretreatment variation in the three individual EASIX parameters at the time of first AML diagnosis also showed a wide variation (Table 2). The platelet levels showed the widest variation, and the relative variation range (i.e., the lowest and the highest values relative to the median level) varied between 0.08 and 10.0. Finally, all three EASIX parameters showed significant correlations with the EASIX score (Spearman’s test, *p* < 0.0002), but the r-values varied between the three parameters with the platelet count having the highest value (Table 2). Thus, the wide variation in the pretreatment EASIX score seemed to be mainly due to the wide variation in pretreatment platelet counts.

The peripheral blood platelet count in AML patients is determined by several factors, including suppression of normal hematopoiesis by marrow-infiltrating AML cells [18,19,20,21] and consumption by coagulopathy [22] or hemophagocytosis [23]. The percentage of AML cells among nucleated bone marrow cells varied between our patients (range 10–99%), but there was no significant correlation between the platelet count and the percentage of AML blasts (Spearman’s test, r = −0.034, *p* = 0.69). However, the EASIX score and the blast percentage showed a weak correlation (Spearman’s test, r = 0.21, *p* = 0.011). Data on coagulopathy and hemophagocytosis are unfortunately not available for our patients.

### 3.3. High EASIX Score Is Associated with Acute Phase Reaction and Monocytic AML Cells

Several studies suggest that detection of an acute phase reaction at the time of first AML diagnosis is associated with an adverse prognosis (reviewed in [24]). We observed a moderate but significant association between pretreatment EASIX and serum CRP level (Spearman’s test, r = 0.24, *p* = 0.002). A weaker association was observed between EASIX score and the alternative parameter CRP/albumin (mg/L) ratio (Spearman’s test, r = 0.23, *p* = 0.006), whereas EASIX score showed no significant correlation with albumin level.

Elderly individuals can have signs of an acute phase reaction with increased CRP levels; this is often referred to as inflammaging [25]. However, even though we observed an association between EASIX score and CRP levels, we did not detect any significant association between EASIX score and age for our AML patients (Spearman’s test, r = 0.039, *p* = 0.62). Furthermore, some previous studies suggest that a high percentage of bone marrow AML blasts is associated with an acute phase reaction (for references and details see [24]. As described above, we observed that the pretreatment EASIX score also showed a significant correlation with the percentage of AML blasts in the bone marrow (Section 3.2).

We divided our patients into two main subsets by using the median EASIX score for the 160 patients as cutoff. The EASIX score ranged from 17 to 483 (median 207) in the low EASIX subset and from 508 to 11,061 (median 1461) in the high EASIX subset. The corresponding log2-transformed values were median 7.7 with range 4.1–8.9 for low EASIX patients and median 10.5 with range 9.0–13.4 for high EASIX patients. When we compared these two contrasting patient subsets, the following observations were made (Table 3):

- Both the high and low EASIX patients included eight patients with secondary AML.
- The difference in percentage of bone marrow blasts between the low (median 54%, range 10–98%) and high EASIX subsets (median 70%, range 15–99%) did not reach statistical significance (Mann–Whitney U-test, *p* = 0.12).
- Monocytic differentiation is an adverse prognostic parameter for AML patients receiving venetoclax-based AML therapy [26,27,28,29]. The high-EASIX group had a significantly increased frequency of patients with monocytic AML blasts, i.e., AML-M4/M5 phenotype according to the WHO 2016 criteria/classification [20], compared with the low-EASIX subset (37 versus 16 patients, Fisher’s test, *p* = 0.0007).
- These two patient subsets did not differ significantly with regard to (i) frequencies of genetic *FLT3* and *NPM1* abnormalities, (ii) frequencies of patients not reaching complete hematological remission after the first induction cycle and (iii) frequency of early death after 2 as well as 3 months. Comparison of karyotype subsets did not reach statistical significance, also reflecting that eight of the 12 monocytic inv(16)/t(16;16) patients showed high EASIX score and represent all patients with a favorable karyotype among the high EASIX patients (see Table 3).

To conclude, we did not observe any significant associations between EASIX score and any strong established prognostic parameters for AML patients receiving intensive anti-AML therapy, i.e., age, genetic abnormalities and response to first induction cycle. However, an increased EASIX score was significantly associated with monocytic AML cell differentiation, which is an adverse prognostic parameter in venetoclax-based AML therapy.

### 3.4. The Pretreatment EASIX Score Shows No Association with Overall Patient Survival After Intensive Anti-AML Therapy

The early mortality of AML patients after 30 days, two months and three months did not differ between the low and high EASIX patient subsets (Table 3). Furthermore, there were no significant differences even when comparing patients in the highest EASIX score quartile versus the other three quartiles (Table 3).

No significant differences in 5-year overall survival were observed when comparing these two main patient subsets (Figure 1 left). We also made an additional comparison of the survival for two contrasting quartiles, i.e., the quartile with the highest versus the quartile with the lowest EASIX scores, and these two quartiles did not differ with regard to 5-year overall survival (Figure 1 right).

### 3.5. Pretreatment EASIX Score Is Not Associated with Overall Posttransplant Survival for AML Patients Receiving Allogeneic Stem Cell Transplantation in First Complete Remission

A total of 65 patients received consolidation therapy including allogeneic stem cell transplantation. The majority of the 65 allotransplanted patients were transplanted in the first complete remission, usually within 3 months after starting induction therapy.

We investigated the overall posttransplant 5-year survival for patients allografted in first complete remission; we then compared the survival for 27 patients with high and 31 patients with low pretherapy (i.e., at diagnosis) EASIX scores (Figure 2). These two subsets did not differ significantly in posttransplant overall survival.

### 3.6. The Overall Survival for Patient Subsets Defined by ELN2022 Criteria for Favorable or Adverse Prognosis: Comparison of Patients with High and Low Pretherapy EASIX Scores

The ELN2022 classification is based on karyotype together with extended mutational analyses not only including *FLT3* and *NPM1* but also *ASXL1*, *BCOR*, *EZH2*, *RUNX1*, *SF3B1*, *SRSF2*, *STAG2*, *U2AF1*, *ZRSR2* and/or *TP53* [19]. Extended mutational analyses were not available for all our patients, and a complete ELN2022 classification was therefore not possible for the whole cohort. However, even the ELN2022 classification allows certain patients to be correctly classified based only on karyotype together with limited mutational analyses. We therefore identify two relatively small patient groups that fulfilled ELN2022 criteria for favorable and adverse prognosis without extended mutational analyses. We would emphasize that these two groups only include a subset of our patients with favorable/adverse ELN2022 prognosis.

Our patients with favorable prognosis according to ELN2022 included patients with favorable karyotypes and patients with non-adverse karyotypes plus normal *FLT3* and mutated *NPM1*. When comparing such patients with high and low pretherapy EASIX scores we observed a difference in borderline significance (Figure 3 left part, *p* = 0.049, high survival in high EASIX patients). It should then be emphasized that the 13 patients with high EASIX scores included 11 patients with monocytic differentiation and among them eight out of the twelve patients with inv(16)/t(16;16) abnormalities.

Our identification of patients with adverse prognosis was based on the ELN2022 karyotype criteria, and these patients did not show any significant differences when comparing overall survival for subsets with high and low pretherapy EASIX (Figure 3 right).

A high EASIX score reflects endothelial/angiogenic dysfunction; such dysfunctions are observed in AML and are then associated with adverse prognosis [1,10,11,12,13,14,15,16,17]. A relevant question is therefore whether high EASIX scores can be used as an easily available biomarker for this dysregulation and thereby reflect its known adverse prognostic impact. The overall results presented in this section are consistent with the conclusion that high EASIX score has no additional adverse prognostic impact for these patients. The higher overall survival for patients with high EASIX scores and favorable prognosis according to ELN2022 (Figure 3 left) reached only borderline significance, and this analysis is based on a small group of patients. The high levels in this group may then be influenced by a coincidental increase in patients showing monocytic AML cell differentiation and not (only) by dysfunctions that have a prognostic impact (see Table 3).

### 3.7. The Pretreatment-Modified EASIX (mEASIX) Score Shows No Association with Overall Patient Survival After Intensive Therapy

The mEASIX score, i.e., a score where the creatinine level is replaced by the serum CRP level, can also be used to predict survival in patients receiving CAR-T cell therapy or allogeneic stem cell transplantation [30,31,32,33]. We therefore calculated the mEASIX score for all our 160 patients. The median mEASIX level was thereafter used as the cutoff to define high- and low-mEASIX patient subsets, but these two subsets did not differ regarding long-term overall survival either (Figure 4).

## 4. Discussion

The EASIX score reflects endothelial activation/dysfunction [2]. Endothelial cells are important both for leukemogenesis and chemosensitivity in human AML: (i) increased bone marrow angiogenesis facilitates the supply of oxygen as well as nutrients to the cell-rich AML bone marrow [13,14,15,16,17]; (ii) the endothelium is also involved in the regulation of cellular trafficking/migration [34]; (iii) endothelial cells support proliferation and survival of AML cells, including the leukemic stem cells in endothelial niches [10,11,12,13]; (iv) angiogenesis/endothelial cells seem important for chemosensitivity and prognosis after intensive and potentially curative chemotherapy [11,16,17]; and (v) endothelial cells are also involved in the development of complications to anti-AML therapy [1,2]. Despite these observations, we could not detect any prognostic impact of the pretherapy EASIX score in our present study of non-APL AML, an observation suggesting that these AML supporting roles of endothelial cells are not reflected in the pretreatment EASIX score. This observation is similar to previous observations in MDS: the EASIX score has a prognostic impact in low-risk MDS, whereas it has no prognostic impact in adverse MDS that has a high risk of AML transformation [7].

The characteristics of our patients are presented in Table 1 and Table S1. In accordance with the age of our patients, they showed an expected variation with regard to genetic abnormalities, frequency of secondary AML, primary refractory disease, early death and expected long-term survival [18,19,35,36,37]. Our patients should thus be regarded as representative of AML in general.

AML is characterized by suppression of normal hematopoiesis by marrow-infiltrating leukemia cells and thereby low levels of circulating normal blood cells [18,19,20,21]. The pretreatment platelet count showed no correlation with the percentage of bone marrow AML blasts in our study; this observation probably reflects that bone marrow suppression is not only determined by the blast percentage but also variation between patients in functional AML cell characteristics, e.g., constitutive release of suppressive soluble mediators. This hypothesis is also consistent with our observation that the pretreatment EASIX score showed only a weak correlation with bone marrow AML cell burden; this weak effect is possibly caused by combined effects of the platelet count and the LD level (i.e., AML cell burden).

Monocytic AML cell differentiation was seen for a subset of our patients when we used the morphological criteria described in the WHO 2016 classification [20]. Such differentiation is not regarded as a prognostic parameter for patients with non-APL variants of AML who receive intensive chemotherapy [19]. However, monocytic differentiation is associated with adverse prognosis in patients receiving BCL2-inhibitory therapy [26,27,28,29] because BCL2 is then less important to support AML cell survival/energy metabolism compared with other AML variants [38]. Monocytic differentiation is also associated with increased constitutive release of several soluble mediators, including chemotactic/angioregulatory mediators [39]. Altered bidirectional crosstalk between AML and endothelial cells may therefore contribute to the increased pretherapy EASIX scores in monocytic AML, but future studies have to clarify whether EASIX scores have any prognostic impact in new targeted therapies, e.g., BCL2-targeting therapy. Finally, AML cells with erythroid/megakaryocytic differentiation show similar resistance to BCL2-targeting therapy [40], but our study included only a few patients with these uncommon AML variants.

A high pretransplant EASIX score has an adverse prognostic impact for patients receiving consolidation therapy with allogeneic stem cell transplantation [1]. In contrast, our present study suggests that the pretreatment EASIX score is not associated with long-term overall survival for non-APL AML patients, including allotransplant recipients. Taken together, these observations indicate that the later pretransplant prognostic EASIX score is (at least partly) independent of the preinduction/pretherapy score. This independence is probably caused by the pretransplant intensive antileukemic therapy with induction of complete hematological remission [19] and normalized peripheral blood platelet counts together with decreased LD levels due to the reduced disease burden.

A previous study described an association between high EASIX score and high early mortality (i.e., death before 30 days) for patients with the APL variant of AML [9]. In contrast, we could not detect a similar association for patients with non-APL variants of AML. The APL study included 131 patients; eight patients died during the first seven days of treatment, and 25 patients died between seven and 30 days after the start of therapy [9]. Most of the 33 APL patients that died during the first 30 days died from complications to APL-associated coagulopathy, i.e., fatal hemorrhage (eight patients), DIC-associated organ failure (eight patients) and thrombosis [9]. Thus, the significant association between high EASIX score and early mortality seems to reflect the APL-induced coagulopathy with endothelial dysfunction. Fatal coagulopathy is relatively uncommon in non-APL variants of AML [21,22], and this is probably the reason why high a EASIX score is significantly associated with early mortality only in APL but not non-APL variants of AML in our present study.

Another previous study described increased early death during the first three months of intensive anti-AML therapy in patients with high pretreatment EASIX scores [41]. We did not observe a similar association between early death and pretreatment EASIX score (see Table 3). Our study had a similar low frequency of early death as the previous study [41], and in our opinion a larger study is needed to finally answer this question of whether pretherapy EASIX score is associated with early death. A possible explanation for the difference between the two studies could be that the patients in the earlier study had a higher median age than our present patients.

The EASIX score in patients receiving allogeneic stem cell transplantation increases early after conditioning/transplantation; after this early increase the score decreases gradually but remains higher than the pretransplant score for a longer time [42]. This early posttransplant increased score then had a stronger prognostic impact than the pretransplant scores. Thus, the EASIX score is not only influenced by the pretransplant status but also by the early posttransplant antileukemic therapy. Furthermore, the pretransplant score is also a post-induction/consolidation therapy score that is probably influenced by a combined effect of several factors, including the pretherapy disease, intensive chemotherapy, induction of complete hematological remission with hematological reconstitution, chemotherapy-induced immunomodulation, reduced AML-induced endothelial modulation because only minimal residual disease remains, and possibly modulation/induction of an acute phase reaction. This is probably the explanation of why we detect no prognostic impact of the initial pretherapy EASIX score whereas the pretransplant EASIX score has a well-documented impact.

We compared the overall survival for patients with high and low pretherapy EASIX scores for two identified groups having favorable and adverse prognosis, respectively, according to the ELN2022 criteria. These results must be interpreted with great care because the groups are small and they included only patients that could be classified according to the ELN2022 criteria without extended mutational analyses [19]. The results also suggest that high pretherapy EASIX scores do not have any additional adverse prognostic impact in these two ELN2022 defined patient groups. The borderline association between high EASIX score and higher survival (*p* = 0.049) in the favorable prognostic group (see Figure 3) is in our opinion rather influenced by an additional and possibly coincidental effect in this group with high EASIX scores caused by monocytic differentiation (see Table 3) that by itself is not regarded as a prognostic parameter in ELN2022 [19]. Finally, our observations suggest that the possible role of monocytes/macrophages behind the development of endothelial dysfunctions/high EASIX should be investigated in other malignancies.

Von Willebrand factor is released by endothelial cells and platelets [43,44]; it is regarded as an acute phase reactant and a marker of endothelial cell activation/dysfunction that is increased in inflammation, e.g., severe acute GVHD after allogeneic stem cell transplantation [45]. A recent study showed that the levels of von Willebrand antigen and von Willebrand:Ristocitin cofactor levels were increased in patients with untreated AML; the levers were then relatively low in AML with a favorable prognosis according to the ELN-2022 classification and higher in patients with adverse prognosis [46]. Both EASIX score (our present study) and von Willebrand factor levels [46] are regarded as acute phase reactants. However, only EASIX score was associated with monocytic AML cell differentiation, whereas only von Willebrand factor levels were associated with coagulopathy/thrombosis/bleeding and age. Previous studies have demonstrated that detection of coagulopathy is independently associated with adverse prognosis in human AML [47]. Thus, even though the EASIX score and von Willebrand factor level show similarities with regard to associations with acute phase reactions, they differ with regard to association with coagulation status. It is not known whether EASIX score and von Willebrand factor levels show any correlation, but even if a correlation is present it would be likely that von Willebrand factor levels have a different (and possibly stronger?) prognostic association due to their association with coagulopathy and karyotype.

Several studies suggest that pretreatment CRP levels have a prognostic impact in human AML [24]. We therefore investigated whether the pretherapy mEASIX score, where creatinine level is replaced by CRP level, had a prognostic impact in AML. The pretherapy mEASIX score seems to have a prognostic impact both for patients receiving CAR-T cell therapy and allotransplant recipients [30,31,32,33], but similar to the EASIX score we did not detect any prognostic impact of the preinduction/pretherapy mEASIX score at the first time of AML diagnosis (see Figure 4).

## 5. Conclusions

Dysfunctional endothelial function and angioregulation are important in leukemogenesis and the development of chemoresistance in human AML [16,17]; experimental studies have also shown that endothelial cells are important for the development of resistance to cytarabine [48] and all our patients received cytarabine-based therapy (see Section 2). The main question addressed in our study is therefore whether the pretherapy EASIX score in patients with first diagnosis of AML is an easily available biomarker that reflects biological characteristics important for leukemogenesis/chemoresistance. We could not find any evidence for using high/increased pretherapy EASIX score as an additional and easily available endothelial biomarker of adverse prognosis in patients with newly diagnosed AML. Possible explanations for this may be that the earliest EASIX score then does not reflect those endothelial characteristics that are important for leukemogenesis/chemoresistance, and/or that the earliest EASIX score, at least for certain patients, is strongly influenced by additional biological characteristics that do not have any significant influence on long-term prognosis for patients receiving intensive chemotherapy, e.g., morphological signs of differentiation (see Table 3) [18,19]. This lack of prognostic association is similar to patients with high-risk MDS [7], a preleukemic disease with a high risk of AML transformation. Whether the EASIX score has a prognostic impact in patients receiving new targeted therapies (e.g., BCL2-targeting) requires further studies to clarify.

## Figures and Tables

**Figure 1:**
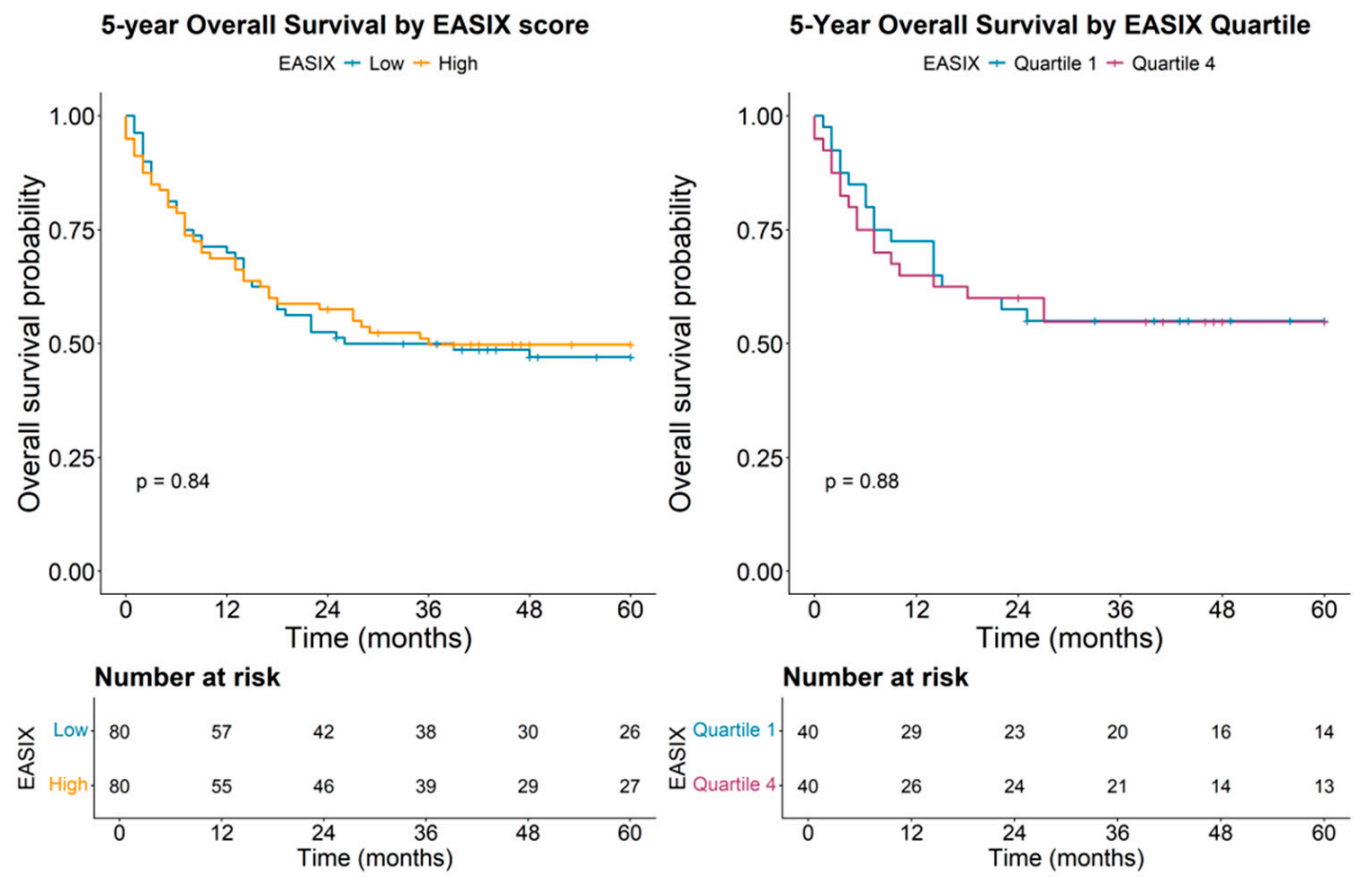
Long-term overall survival curves (Kaplan–Meier analyses): A comparison of AML patient subsets with high and low preinduction EASIX scores. (**Left**) This figure shows the overall survival curves when the 160 patients were divided into two subsets referred to as the high and low EASIX subsets (i.e., cutoff being the cohort median score of 495, the log2-transformed median being 9.0). (**Right**) The figure presents the curves for the two contrasting patient quartiles with the highest (quartile 4) and lowest (quartile 1) EASIX scores (40 patients in each quartile).

**Figure 2:**
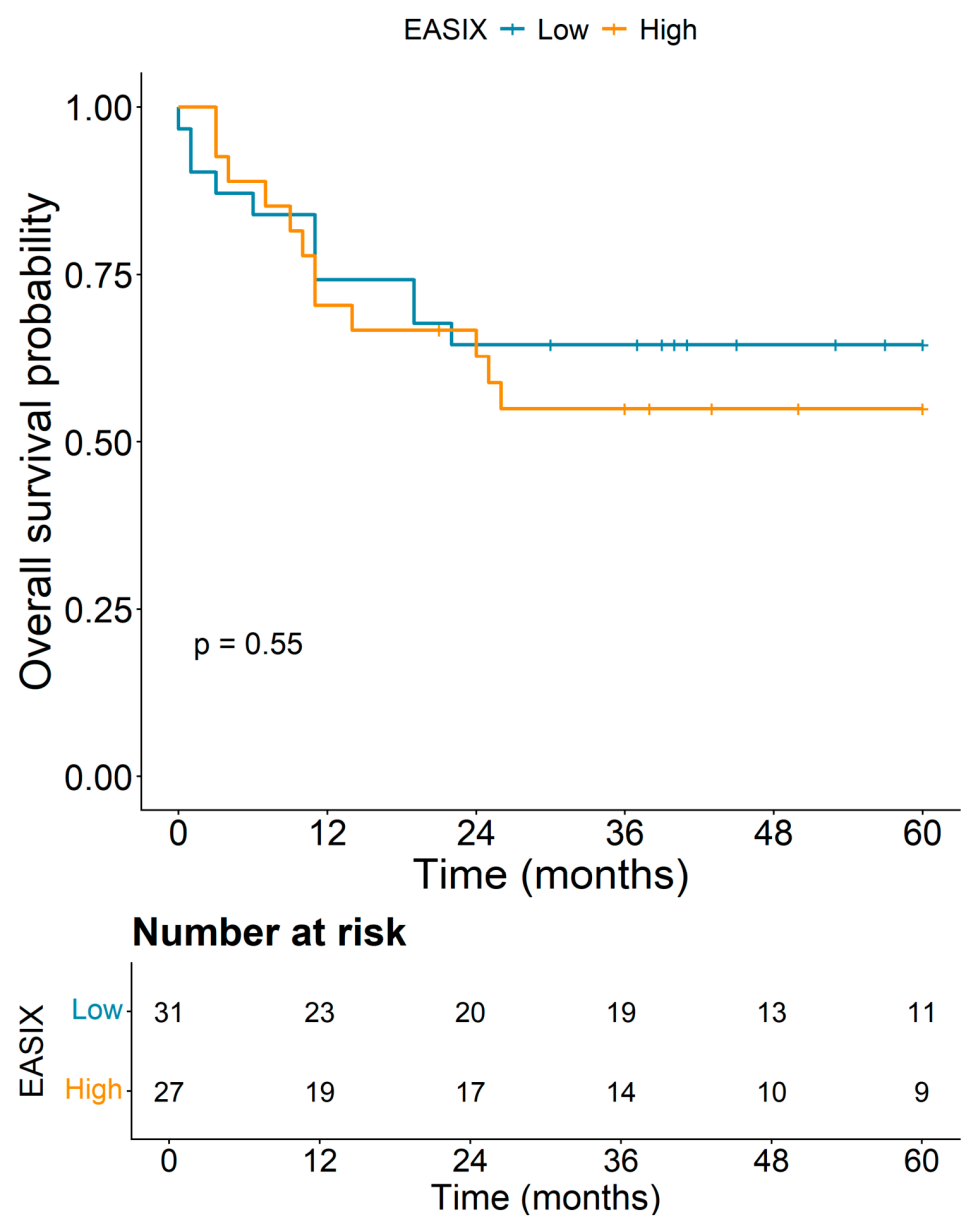
Long-term overall posttransplant survival curves (Kaplan–Meier analyses): An analysis based on 58 patients allotransplanted in first complete remission. Patients were classified according to a high or low preinduction EASIX scores, the cutoff then being the whole cohort median score of 495 (log2-transformed median 9.0).

**Figure 3:**
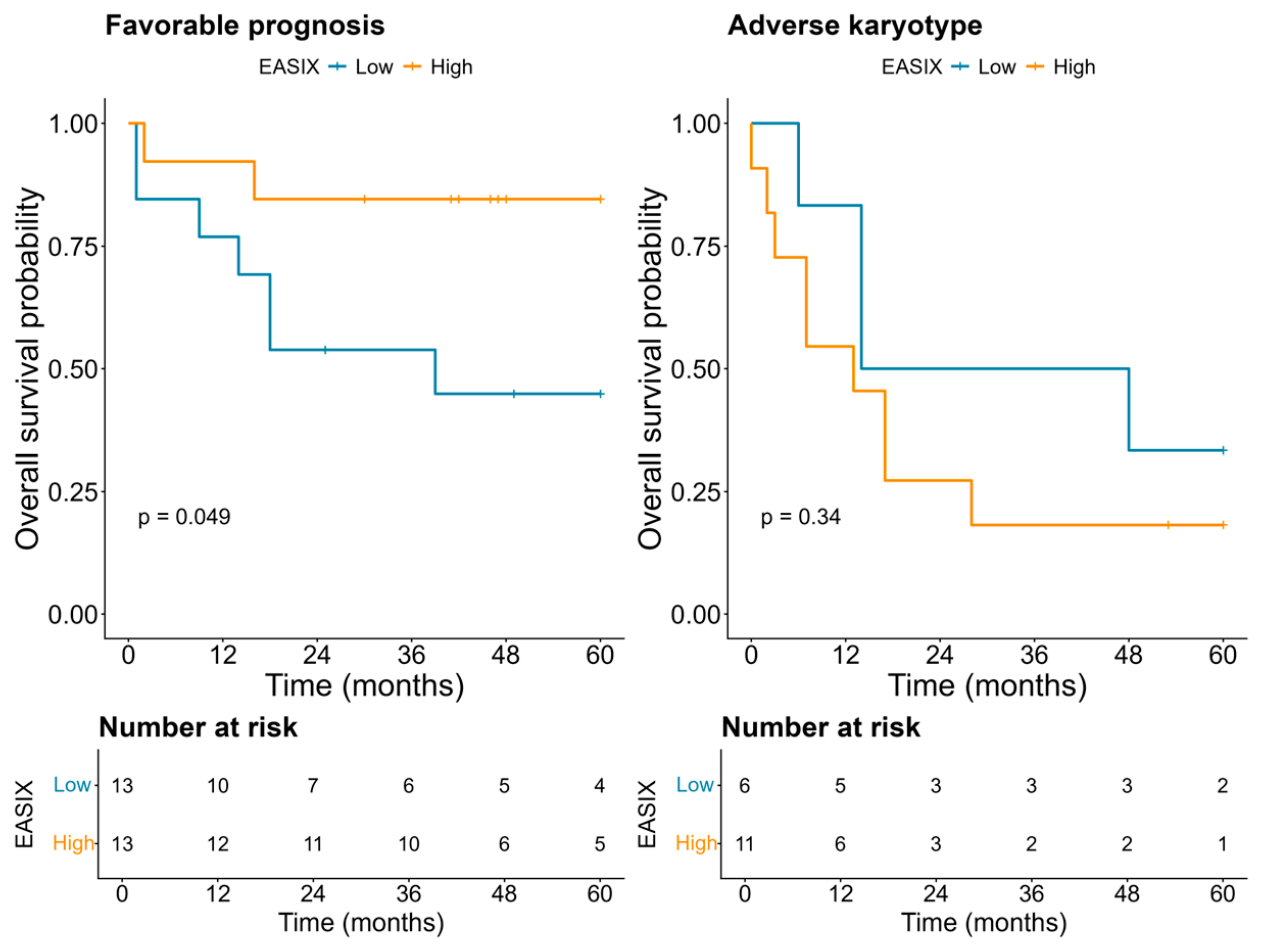
Long-term overall survival curves (Kaplan–Meier analyses): A comparison of AML patient subsets identified according to the ELN2022 criteria and showing either high or low preinduction EASIX scores. (**Left**) This figure shows the overall survival curves for 26 patients with favorable prognosis and having either high (*n* = 13) or low (*n* = 13) pretherapy EASIX scores (i.e., EASIX scores being either higher or lower than the cohort median score of 495 (log2-transformed median 9.0), respectively). (**Right**) The figure presents the curves for patients with adverse prognosis and having either high (11 patients) or low (6 patients) pretherapy EASIX scores.

**Figure 4:**
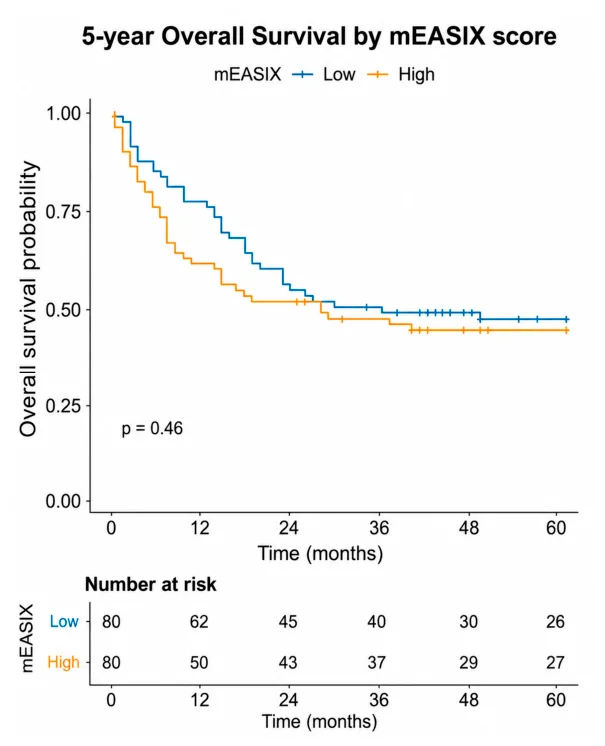
Long-term overall survival curves (Kaplan–Meier analyses): A comparison of AML patient subsets with high and low pretreatment-modified EASIX (mEASIX) scores. This figure shows the overall survival curves when the 160 patients were divided into two subsets referred to as the high- and low-mEASIX subsets (i.e., mEASIX scores being either higher or lower than the median cohort score, respectively).

**Table 1:** Clinical and biological characteristics of the 160 AML patients included in the study.

Characteristic	*n* = 160
Sex (number of males/females)	98/62
Age (years, median and range)	55 (19–79)
Secondary AML (number)	16
Pretreatment CRP (mg/L, median and range)	28 (1–419)
Bone marrow blasts at first diagnosis (%, median and range)	65 (10–99)
Monocytic AML cell differentiation (i.e., number with FAB M4/M5) [20]	53
Classification of AML karyotype (numbers) [18,19]	
Favorable	12
Normal	98
Intermediate	26
Adverse	17
Unknown	7
*FLT3-ITD* (number)	47
*NPM1* mutations (number)	33
Patients with favorable genetic abnormalities defined as Normal karyotype + no *FLT3* mutation + *NPM1* mutation [19]	14
Remission after first induction cycle (number)	124
Death with primary resistant disease (number)	9
Death during the first 30 days after start of therapy (number)	8
Death first 2 months after initiation of intensive therapy (number)	10
Death first 3 months after initiation of intensive therapy (number)	17
Death in complete remission among these patients (number)	8
Death without reaching remission (number)	9

**Table 2:** The pretreatment levels of individual parameters used to calculate the EASIX and the mEASIX scores, i.e., peripheral blood platelet count, serum LD level, serum creatinine level and serum CRP level. The table presents a summary of the results for all 160 patients included in the present study. For each parameter, the table presents the median level and the range together with the relative range (i.e., defined as the lowest and highest levels relative to the median level) and the correlation between the parameter levels and EASIX score. The correlation between a parameter and the EASIX score was investigated using Spearman’s correlation test, and these results are presented as the r-value together with the corresponding *p*-value in parentheses (right column).

Parameter	Median	Range (Relative Range)	EASIX Correlation r-Value (*p*-Value)
Platelet count (×10^9^/L)	62	5–632 (0.08–10.0)	0.77 (<0.0002)
LD (IU/L)	380	129–6123 (0.34–16.1)	0.59 (<0.0002)
Creatinine (µmol/L)	76	35–206 (0.46–2.71)	0.29 (<0.0002)
CRP (mg/L)	28	1–419 (0.04–15.0)	0.24 (0.002)

**Table 3:** The characteristics of the two patient subsets with high (exceeding the median EASIX score) and low (below the median) EASIX scores. The EASIX scores are presented as the absolute calculated values. The results from the comparison of the two patient subsets are presented in the right columns. Continuous data were then analyzed by the Mann–Whitney U test and categorized data by Fisher’s exact test except for the karyotype data that were analyzed by the Chi-Square test.

Characteristic	Low EASIX Score (*n* = 80)	High EASIX Score (*n* = 80)	*p*-Value
EASIX score (median and range)	207 (17–483)	1461 (508–11,061)	
Log2 transformed EASIX score (median and range)	7.7 (4.1–8.9)	10.5 (9.0–13.4)	
CRP (mg/L; median and range)	22 (1–281)	37 (1–419)	0.017
Bone marrow blasts (% of nucleated cells)	61 (10–98)	70 (15–99)	0.12
Monocytic AML cell differentiation (number).	16	37	0.0007
Death first 30 days (patient number)	3	7	0.33
Favorable//normal/intermediate/adverse karyotype (number)	4/53/14/6	8/45/12/11	0.31
*Flt3*-ITD (number)	21	26	0.38
*NPM1* mutation (number)	18	15	0.54
Death first 2 months (patient number)	3 (1 + 2) *	7 (4 + 3)	0.33
Death first 3 months (patient numbers)	8 (3 + 5) *	9 (5 + 4)	1.00
Remission after first induction cycle (number)	65	57	0.19

* Parentheses state the numbers for the corresponding quartiles (i.e., low quartiles 1 and 2 + high quartiles 3 and 4).

## Data Availability

The raw data supporting the conclusions of this article will be made available by the authors on request.

## Supplementary Materials

The following supporting information can be downloaded at: https://www.mdpi.com/article/10.3390/diagnostics16193117/s1, Table S1: Key information for each individual patient included in the study; the basis for detection of statistically significant associations/differences and calculations of EASIX/mEASIX; Table S2: EASIX and mEASIX scores for individual patients: a presentation of the calculated and the log2 transformed pretreatment scores.

## Abbreviations

The following abbreviations are used in this manuscript:

AML	Acute myeloid leukemia
APL	Acute promyelocytic leukemia
BCL2	B cell lymphoma 2
CAR-T cell	Chimeric antigen receptor T cell
CRP	C reactive protein
EASIX	Endothelial activation and stress index
FAB	French–American–British
FLT3	FMS-like tyrosine kinase 3
ITD	Internal tandem duplication
LD	Lactate dehydrogenase
MDS	Myelodysplastic syndrome
mEASIX	Modified endothelial activation and stress index
NPM1	Nucleophosmin 1
WHO	World Health Organization

## References

[1] Reikvam H., Hatfield K., Sandnes M., Bruserud Ø. (2025). Future biomarkers for acute graft-versus-host disease: Potential roles of nucleic acids, metabolites, and immune cell markers. Expert Rev. Clin. Immunol..

[2] Simons L., Alasfar L., Qadoura M., Buhl J., Sunderer F., Korell F., Ikonomidis I., Dietrich M., Seidlitz S., Vink H. (2025). Comprehensive assessment of endothelial dysfunction before cellular therapy: EASIX, local imaging, and systemic biomarkers. Blood Vessel. Thromb. Hemost..

[3] Xia Y., Liang A., Wang M., Zhang J. (2025). Risk analysis of the association between EASIX and all-cause mortality in critical ill patients with atrial fibrillation: A retrospective study from MIMIC-IV database. Eur. J. Med. Res..

[4] Finke D., Hund H., Frey N., Luft T., Lehmann L.H. (2025). EASIX (endothelial activation and stress index) predicts mortality in patients with coronary artery disease. Clin. Res. Cardiol..

[5] Li N., Yin J., Zeng Y., Li S., Huang C., Chen G., Luo H., Jiang Y. (2025). Association between endothelial activation and stress index and all-cause mortality risk in sepsis patients: A retrospective cohort analysis utilizing the MIMIC-IV database. BMC Infect. Dis..

[6] Gu J.S., Ryu J.W., Yu S.H., Chung H.S., Hwang J.E., Bae W.K., Ku J.Y., Lee C.H., Ha H.K., Jung S.I. (2022). Prognostic value of the endothelial activation and stress index in patients with upper tract urothelial cancer undergoing radical nephroureterectomy. Investig. Clin. Urol..

[7] Merz A., Germing U., Kobbe G., Kaivers J., Jauch A., Radujkovic A., Hummel M., Benner A., Merz M., Dreger P. (2019). EASIX for prediction of survival in lower-risk myelodysplastic syndromes. Blood Cancer J..

[8] Park S., Go S.I., Lee G.W. (2022). The Endothelial Activation and Stress Index (EASIX) score is an independent prognostic factor in patients with diffuse large B-cell lymphoma. BMC Cancer.

[9] Hunutlu F., Özkocaman V., Baysal M., Öztop H., Güllülü Boz S., Ada Tak N., Sertkaya O., Kara İ., Akar E., Çakmak Ş. (2026). The Dynamic Endothelial Activation and Stress Index (EASIX) as a Predictor of Early Death and Long-Term Survival in Acute Promyelocytic Leukemia (APL): A Multicenter Study. Cancers.

[10] Fodil S., Arnaud M., Vaganay C., Puissant A., Lengline E., Mooney N., Itzykson R., Zafrani L. (2022). Endothelial cells: Major players in acute myeloid leukaemia. Blood Rev..

[11] Wang B., Wang X., Hou D., Huang Q., Zhan W., Chen C., Liu J., You R., Xie J., Chen P. (2019). Exosomes derived from acute myeloid leukemia cells promote chemoresistance by enhancing glycolysis-mediated vascular remodeling. J. Cell Physiol..

[12] Hatfield K., Ryningen A., Corbascio M., Bruserud Ø. (2006). Microvascular endothelial cells increase proliferation and inhibit apoptosis of native human acute myelogenous leukemia blasts. Int. J. Cancer.

[13] Behrmann L., Wellbrock J., Fiedler W. (2018). Acute Myeloid Leukemia and the Bone Marrow Niche-Take a Closer Look. Front. Oncol..

[14] Hussong J.W., Rodgers G.M., Shami P.J. (2000). Evidence of increased angiogenesis in patients with acute myeloid leukemia. Blood.

[15] Padró T., Ruiz S., Bieker R., Bürger H., Steins M., Kienast J., Büchner T., Berdel W.E., Mesters R.M. (2000). Increased angiogenesis in the bone marrow of patients with acute myeloid leukemia. Blood.

[16] Reddy V., Moreb J. (2000). Prognostic correlation of increased angiogenesis in acute myeloid leukemia with cytogenetics. Blood.

[17] Shih T.T., Hou H.A., Liu C.Y., Chen B.B., Tang J.L., Chen H.Y., Wei S.Y., Yao M., Huang S.Y., Chou W.C. (2009). Bone marrow angiogenesis magnetic resonance imaging in patients with acute myeloid leukemia: Peak enhancement ratio is an independent predictor for overall survival. Blood.

[18] Döhner H., Estey E., Grimwade D., Amadori S., Appelbaum F.R., Büchner T., Dombret H., Ebert B.L., Fenaux P., Larson R.A. (2017). Diagnosis and management of AML in adults: 2017 ELN recommendations from an international expert panel. Blood.

[19] Döhner H., Wei A.H., Appelbaum F.R., Craddock C., DiNardo C.D., Dombret H., Ebert B.L., Fenaux P., Godley L., Hasserjian R.P. (2022). Diagnosis and management of AML in adults: 2022 recommendations from an international expert panel on behalf of the ELN. Blood.

[20] Arber D.A., Orazi A., Hasserjian R., Thiele J., Borowitz M.J., Le Beau M.M., Bloomfield C.D., Cazzola M., Vardiman J.W. (2016). The 2016 revision to the World Health Organization classification of myeloid neoplasms and acute leukemia. Blood.

[21] Kusuda M., Nakasone H., Nakamura Y., Kawamura M., Takeshita J., Kawamura S., Yoshino N., Misaki Y., Yoshimura K., Matsumi S. (2023). Very early death within 30 days after diagnosis in patients with acute myeloid leukemia. Int. J. Hematol..

[22] Libourel E.J., Klerk C.P.W., van Norden Y., de Maat M.P.M., Kruip M.J., Sonneveld P., Löwenberg B., Leebeek F.W.G. (2016). Disseminated intravascular coagulation at diagnosis is a strong predictor for thrombosis in acute myeloid leukemia. Blood.

[23] Delavigne K., Bérard E., Bertoli S., Corre J., Duchayne E., Demur C., Mansat-De Mas V., Borel C., Picard M., Alvarez M. (2014). Hemophagocytic syndrome in patients with acute myeloid leukemia undergoing intensive chemotherapy. Haematologica.

[24] Reikvam H., Rolfsnes M.G., Rolsdorph L., Sandnes M., Selheim F., Hernandez-Valladares M., Bruserud Ø. (2025). Ferritin in Acute Myeloid Leukemia: Not Only a Marker of Inflammation and Iron Overload, but Also a Regulator of Cellular Iron Metabolism, Signaling and Communication. Int. J. Mol. Sci..

[25] Franceschi C., Garagnani P., Parini P., Giuliani C., Santoro A. (2018). Inflammaging: A new immune-metabolic viewpoint for age-related diseases. Nat. Rev. Endocrinol..

[26] Pei S., Pollyea D.A., Gustafson A., Stevens B.M., Minhajuddin M., Fu R., Riemondy K.A., Gillen A.E., Sheridan R.M., Kim J. (2020). Monocytic Subclones Confer Resistance to Venetoclax-Based Therapy in Patients with Acute Myeloid Leukemia. Cancer Discov..

[27] Pei S., Shelton I.T., Gillen A.E., Stevens B.M., Gasparetto M., Wang Y., Liu L., Liu J., Brunetti T.M., Engel K. (2023). A Novel Type of Monocytic Leukemia Stem Cell Revealed by the Clinical Use of Venetoclax-Based Therapy. Cancer Discov..

[28] White B.S., Khan S.A., Mason M.J., Ammad-Ud-Din M., Potdar S., Malani D., Kuusanmäki H., Druker B.J., Heckman C., Kallioniemi O. (2021). Bayesian multi-source regression and monocyte-associated gene expression predict BCL-2 inhibitor resistance in acute myeloid leukemia. npj Precis. Oncol..

[29] Zhao L., Yang J., Chen M., Xiang X., Ma H., Niu T., Gong Y., Chen X., Liu J., Wu Y. (2024). Myelomonocytic and monocytic acute myeloid leukemia demonstrate comparable poor outcomes with venetoclax-based treatment: A monocentric real-world study. Ann. Hematol..

[30] Cui K., Zhang S., Du Y., Wang Q., Chai Y., Gao L., Hu Y., Li B., Tian Y., Zhang Y. (2026). Modified EASIX score on day 7 predicts survival and non-relapse mortality in pediatric acute leukemia undergoing haploidentical stem cell transplantation. Front. Immunol..

[31] Pennisi M., Sanchez-Escamilla M., Flynn J.R., Shouval R., Alarcon Tomas A., Silverberg M.L., Batlevi C., Brentjens R.J., Dahi P.B., Devlin S.M. (2021). Modified EASIX predicts severe cytokine release syndrome and neurotoxicity after chimeric antigen receptor T cells. Blood Adv..

[32] Zhao Y., Zhang X., Zhang M., Guo R., Zhang Y., Pu Y., Zhu H., Liu P., Zhang Y., He X. (2024). Modified EASIX scores predict severe CRS/ICANS in patients with acute myeloid leukemia following CLL1 CAR-T cell therapy. Ann. Hematol..

[33] Yanık A., Sadri S., Şan A., Gül Ö., Koca T., Hunutlu F., Bilen Y., Gürsoy V., Özkocaman V., Özkalemkaş F. (2026). Time-Dependent Prognostic Value of the mEASIX Score in Patients with Myelodysplastic Syndromes. Diagnostics.

[34] Ley K., Laudanna C., Cybulsky M.I., Nourshargh S. (2007). Getting to the site of inflammation: The leukocyte adhesion cascade updated. Nat. Rev. Immunol..

[35] Abrahão R., Keogh R.H., Lichtensztajn D.Y., Marcos-Gragera R., Medeiros B.C., Coleman M.P., Ribeiro R.C., Keegan T.H. (2016). Predictors of early death and survival among children, adolescents and young adults with acute myeloid leukaemia in California, 1988-2011: A population-based study. Br. J. Haematol..

[36] Ho G., Jonas B.A., Li Q., Brunson A., Wun T., Keegan T.H.M. (2017). Early mortality and complications in hospitalized adult Californians with acute myeloid leukaemia. Br. J. Haematol..

[37] Percival M.E., Tao L., Medeiros B.C., Clarke C.A. (2015). Improvements in the early Death rate among 9380 patients with acute myeloid leukemia after initial therapy: A SEER database analysis. Cancer.

[38] Bruserud Ø., Selheim F., Hernandez-Valladares M., Reikvam H. (2024). Monocytic Differentiation in Acute Myeloid Leukemia Cells: Diagnostic Criteria, Biological Heterogeneity, Mitochondrial Metabolism, Resistance to and Induction by Targeted Therapies. Int. J. Mol. Sci..

[39] Brenner A.K., Tvedt T.H., Nepstad I., Rye K.P., Hagen K.M., Reikvam H., Bruserud Ø. (2017). Patients with acute myeloid leukemia can be subclassified based on the constitutive cytokine release of the leukemic cells; the possible clinical relevance and the importance of cellular iron metabolism. Expert Opin. Ther. Targets.

[40] Kuusanmäki H., Dufva O., Vähä-Koskela M., Leppä A.M., Huuhtanen J., Vänttinen I., Nygren P., Klievink J., Bouhlal J., Pölönen P. (2023). Erythroid/megakaryocytic differentiation confers BCL-XL dependency and venetoclax resistance in acute myeloid leukemia. Blood.

[41] Finotto T., Tauveron-Jalenques U., Barrière S., Adelou S., Futier E. (2026). EASIX score is predictive of early fatal complications in newly diagnosed acute myeloid leukemia. Ann. Hematol..

[42] Nawas M.T., Sanchez-Escamilla M., Devlin S.M., Maloy M.A., Ruiz J.D., Sauter C.S., Giralt S.A., Perales M.A., Scordo M. (2022). Dynamic EASIX scores closely predict nonrelapse mortality after allogeneic hematopoietic cell transplantation. Blood Adv..

[43] Sang Y., Roest M., de Laat B., de Groot P.G., Huskens D. (2021). Interplay between platelets and coagulation. Blood Rev..

[44] Reinders J.H., de Groot P.G., Sixma J.J., van Mourik J.A. (1988). Storage and secretion of von Willebrand factor by endothelial cells. Haemostasis.

[45] Martinez-Sanchez J., Charry P., Moreno-Castaño A.B., Ramos A., Torramade-Moix S., Ventosa-Capell H., Palomo M., Penack O., Salas M.Q., Suárez-Lledó M. (2026). Endothelial cell damage in patients with acute graft versus host disease receiving treatment with extracorporeal photopheresis. Front. Immunol..

[46] El-Sayed H.A., Sakr D.H., Ebrahim M.A., Alghandour R., Eid E., Denewer M., Saarsalu B., Othman M., Azzam H. (2026). Von Willebrand, factor VIII, and D-dimer levels as predictors of risk and clinical outcomes in acute myeloid leukemia. Clin. Exp. Med..

[47] Martella F., Cerrano M., Di Cuonzo D., Secreto C., Olivi M., Apolito V., D’Ardia S., Frairia C., Giai V., Lanzarone G. (2022). Frequency and risk factors for thrombosis in acute myeloid leukemia and high-risk myelodysplastic syndromes treated with intensive chemotherapy: A two centers observational study. Ann. Hematol..

[48] Okamoto S., Miyano K., Kitakaze K., Kato H., Yamauchi A., Kajikawa M., Itsumi M., Kawai C., Kuribayashi F. (2022). Coculture in vitro with endothelial cells induces cytarabine resistance of acute myeloid leukemia cells in a VEGF-A/VEGFR-2 signaling-independent manner. Biochem. Biophys. Res. Commun..

