# Supplementary material for: The Pretherapy Endothelial Activation and Stress Index (EASIX) in Patients with Newly Diagnosed Acute Myeloid Leukemia: A Pilot Study
Source: Diagnostics (Basel). 2026 Sep 25;16(19):3117. doi: 10.3390/diagnostics16193117 (PMC13664111; doi:10.3390/diagnostics16193117)
Supplement: Supplementary file 1 [file diagnostics-16-03117-s001.zip › diagnostics-4413465-supplementary.pdf]

TABLE S1 - Key information for each individual patient included in the study; the basis for detection of statistically significant associations/differences and calculations of EASIX/mEASIX.

The table presents the key characteristics for each of the 160 patients included in the present study. The table includes the patient id-number, age at the time of first diagnosis, patients with monocytic differentiation of the AML cells, the bone marrow blast infiltration presented as the percentage among nucleated cells in the bone marrow, the karyotype presented together with genetic *FLT3* (internal tandem duplications, ITD; D836 mutation) and *NPM1* mutations, patients receiving consolidation therapy including allogeneic stem cell transplantation in complete remission together with survival (months), whether the patient is dead (1) or still alive (0) at the time of last follow-up, and the pretherapy/preinduction laboratory values for calculation of EASIX and modified EASIX. The calculated and log2-transformed EASIX scores are presented in the two right columns.

Abbreviations: CRP, C-reactive protein; INS, insertion; ITD, Internal tandem duplication; LD, lactate dehydrogenase; SCT, stem cell transplantation

Patient ID number	Age at diagnosis (years)	Monocytic AML cell morphology	Bone marrow blasts, % of nucleated cells	Karyotype	FLT3	NPM1	Allo-SCT	Survival, months	Death, marked with 1	CRP	LD	Creatinine	Platelets	EASIX score (calculated)	EASIX score log2 transformed
2	60	yes	83	Normal	ITD	INS		171	0	22	409	108	308	143	7.2
3	37		69	Normal	ITD		Allo-SCT	192	0	4	142	68	252	38	5.2
4	64	yes	33	Normal		INS		14	1	28	207	71	62	237	7.9
5	60		30	Complex			Allo-SCT	6	1	149	294	56	173	95	6.6
6	59	yes	50	47, XY, +8				4	1	5	2659	135	45	7977	13.0
7	34		80	Normal			Allo-SCT	197	0	8	352	70	13	1895	10.9
9	66		70	Normal	ITD		Allo-SCT	6	1	125	621	61	49	773	9.6

10	43		50	-3	ITD		Allo-SCT	142	0	28	134	59	102	78	6.3
11	45		24	-7				17	1	14	443	159	129	546	9.1
12	53		88	Normal				36	1	274	465	44	32	639	9.3
13	33	yes	82	Normal	ITD	INS	Allo-SCT	14	1	86	816	87	23	3086	11.6
14	32		41	Normal				102	0	19	888	48	125	340	8.4
15	51		56	Normal				152	0	<1	206	84	66	262	8.0
17	63		91	-Y	ITD			169	0	186	334	121	21	1924	10.9
19	32		90	Normal			Allo-SCT	203	0	103	791	66	42	1243	10.3
20	20		89	Normal				107	0	<1	130	70	277	33	5.0
21	54			Normal	ITD	INS	Allo-SCT	14	1	112	901	42	233	162	7.3
22	46		70	i(13)			Allo-SCT	29	1	2	283	64	26	696	9.4
24	65		37	Normal		INS		1	1	115	389	56	94	232	7.9
25	37		85	Normal	ITD			87	0	1	222	69	74	207	7.7
26	69		33	Normal				23	1	5	185	79	25	584	9.2
28	69			-Y				12	1	7	134	90	38	317	8.3
29	68		20	Normal	ITD	INS		16	1	26	389	65	37	683	9.4
31	48	yes	30	Normal			Allo-SCT	99	0	4	522	76	11	3607	11.8
32	74		68	Normal				7	1	189	656	86	22	2564	11.3
33	40		90	Normal	ITD			111	0	9	247	48	73	162	7.3
34	32		90	Normal			Allo-SCT	126	1	73	263	35	30	307	8.3
34	49		30	-7			Allo-SCT	92	0	2	311	71	206	107	6.7
36	61	yes	40	Normal				17	1	8	163	65	32	331	8.4
37	62	yes	87	Normal	ITD	INS		3	1	125	715	62	19	2333	11.2
38	19	yes	61	Normal			Allo-SCT	109	0	10	785	153	69	1741	10.8
39	28	yes	27	Normal				120	0	35	374	96	54	665	9.4
40	65	yes	69	Normal				8	1	177	396	98	65	597	9.2
41	32		94	Normal				87	0	4	930	94	9	9713	13.2
43	69			Normal				87	0	28	658	114	96	781	9.6
44	52		25	Normal			Allo-SCT	33	0	5	144	86	122	102	6.7
46	19	yes		Normal			Allo-SCT	69	0	57	452	62	35	801	9.6

48	62		95	Normal	ITD		Allo-SCT	10	1	173	896	99	29	3058	11.6
51	54	yes	50	Normal	ITD	INS		78	0	36	986	67	47	1406	10.5
53	58		15	Normal			Allo-SCT	22	1	27	302	88	55	483	8.9
55	41			Normal	ITD			74	0	9	771	89	14	4901	12.3
56	75			Normal		INS		9	1	30	313	92	121	238	7.9
57	65		32	45, X, -X /46, XX				84	0	91	374	65	98	248	8.0
58	58	yes	20	Normal			Allo-SCT	65	0	62	193	76	82	179	7.5
60	46	yes	80	inv(16)				231	0	212	594	91	25	2162	11.1
62	65		54	Normal		INS	Allo-SCT	60	0	99	150	82	180	68	6.1
64	68		70	Normal				66	0	13	193	73	81	173	7.4
65	44	yes	47	inv(16) + 22				63	0	198	782	101	44	1795	10.8
66	49		35	Normal			Allo-SCT	14	1	160	483	76	43	853	9.7
67	65		23	Normal			Allo-SCT	65	0	13	200	64	53	242	7.9
70	71		20	Normal				65	1	10	195	69	14	961	9.9
71	29	yes	92	Normal	ITD		Allo-SCT	185	0	28	1618	78	96	1314	10.4
72	60		86	-7				7	1	6	208	92	27	708	9.5
73	69		38	Normal			Allo-SCT	2	1	23	1290	64	340	243	7.9
74	39		85	Normal			Allo-SCT	44	0	117	261	99	178	145	7.2
75	46		99	Normal	ITD		Allo-SCT	218	0	38	477	63	8	3654	11.8
76	41	yes		Normal	ITD	INS	Allo-SCT	39	0	91	2948	96	119	2378	11.2
77	55		26	Normal			Allo-SCT	22	1	3	129	65	108	78	6.3
78	57		10	-7			Allo-SCT	14	1	12	282	73	187	102	6.7
79	41		46	+8			Allo-SCT	60	0	3	148	82	154	79	6.3
80	22		45	Normal	ITD		Allo-SCT	67	0	203	436	76	123	269	8.1
81	65		90	Normal		INS		25	0	281	365	46	84	200	7.6
82	49	yes	35	Normal				5	1	141	295	77	85	267	8.1
83	63		45	Normal			Allo-SCT	56	0	4	135	75	147	69	6.1
84	41		78	Normal			Allo-SCT	48	0	45	282	49	45	307	8.3
86	30		36	inv(16)				48	0	4	666	118	42	1871	10.9
87	21		96	Normal	ITD		Allo-SCT	46	0	23	1256	49	44	1399	10.5

88	53	yes	90	Normal				236	0	102	280	109	30	1017	10.0
90	53	yes	93	Normal	ITD	INS	Allo-SCT	24	0	243	327	96	11	2853	11.5
91	67	yes	44	-7			Allo-SCT	17	1	14	646	99	81	789	9.6
92	59		20	57-61, XX;+1,+2,+3,-5,+6,+8,+9,-10,+13,+14,+15,+18,+19,+20+,+21,+22, +2~5der			Allo-SCT	13	1	8	293	75	21	1046	10.0
93	44		50	+22, inv(16)				30	0	24	501	81	45	902	9.8
94	32		62	inv(16)			Allo-SCT	229	0	33	383	55	80	263	8.0
96	60		25	+8				9	1	1	404	66	8	3333	11.7
97	30	yes	55	Normal				223	0	3	356	57	248	82	6.4
98	67		56	Normal				3	1	11	321	79	71	357	8.5
99	44	yes	47	inv(16) + 22				63	0	117	746	120	24	3730	11.9
100	52		30					26	1	4	206	66	43	316	8.3
101	22	yes	83	47, XX, +8, t(9;11)				17	1	35	299	59	57	310	8.3
102	64		90	45, XX, -7				265	0	45	3004	106	45	7076	12.8
103	55	yes	40	46, XX, inv(16)			Allo-SCT	53	0	18	495	75	73	508	9.0
104	55	yes	95	Normal	ITD	INS		0	1	99	534	83	51	869	9.8
106	45		25	Normal				35	1	10	176	85	22	680	9.4
108	70	yes	82	Normal				18	1	81	531	58	16	1925	10.9
111	42			inv(16)			Allo-SCT	112	1	3	295	82	327	74	6.2
112	67		90	-5	ITD			48	1	33	146	90	51	258	8.0
113	54		38	Normal		INS		49	0	179	435	67	159	183	7.5
114	66	yes		t(16;16)/47, XX, +22				1	1	14	717	98	35	201	7.7
116	60		80	Normal	ITD			3	1	276	280	65	49	371	8.5
117	40	yes		Inv(16)				46	0	9	582	94	31	1764	10.8
118	49		98	Normal				250	0	10	380	71	34	794	9.6
119	60	yes		Normal	ITD			7	1	22	1769	93	150	19	4.2
120	41	yes	70	Normal				211	0	61	262	59	142	1097	10.1
125	33		85	Normal			Allo-SCT	84	1	84	198	75	49	303	8.2
126	64	yes	70	Complex				3	1	66	2451	147	46	7832	12.9

127	60			-5				7	1	122	284	54	5	3067	11.6
128	33	yes		Inv(16)(p13q22)				41	0	85	775	93	48	1501	10.6
129	70	yes	84	+8				22	1	6	329	120	119	332	8.4
131	48	yes	85	Normal	ITD		Allo-SCT	202	0	1	254	75	237	80	6.3
132	67	yes	65	45-48XY …	ITD		Allo-SCT	4	1	275	445	60	173	154	7.3
133	23		20	del(1;7)(q10p10), +1[11]/46XX[9]			Allo-SCT	43	0	24	175	60	163	64	6.0
134	44		25	+8(18)/46XY(2)			Allo-SCT	40	0	<1	231	67	273	57	5.8
136	69			Normal				0	1	419	2334	109	23	11061	13.4
137	61	yes	92	t(9;11)(p21,3,q23,3)[20]			Allo-SCT	7	1	33	1960	64	207	606	9.2
138	66		30	Normal				3	1	2	546	77	360	117	6.9
139	68			Normal				2	1	3	168	103	47	368	8.5
140	35		80		ITD			27	1	111	1219	89	37	2932	11.5
141	54	yes	50	Normal	ITD	INS		77	0	36	986	67	47	1405	10.5
142	49	yes	40	del Y		INS		42	0	13	1097	70	136	547	9.1
143	62	yes	82	t(6,11)(q27;q23),	ITD		Allo-SCT	28	1	136	511	115	73	805	9.7
145	73		90	Normal	ITD	INS		40	0	108	259	81	94	223	7.8
147	29		98	Normal	ITD		Allo-SCT	42	0	15	489	75	104	353	8.5
148	62	yes	90	Normal	D836	INS		39	0	19	6123	58	63	5637	12.5
149	63		95	Normal	ITD	INS		37	0	10	2068	74	395	387	8.6
151	39		85	del(7)(q22)(19)/46,XY			Allo-SCT	44	0	117	261	99	178	145	7.2
152	61		22	+13[19]/46XY[1]			Allo-SCT	41	0	1	485	92	24	1859	10.9
154	72		80					2	1	45	182	72	94	139	7.1
155	30	yes	75	Inv(16)				47	0	9	582	94	31	1764	10.8
156	41	yes		Normal	ITD	INS	Allo-SCT	39	0	86	2948	93	103	2661	11.4
157	63		22	Normal				13	1	15	299	115	127	234	7.9
158	46		21	-Y				181	0	46	489	66	102	316	8.3
159	53		80	+13			Allo-SCT	170	0	9	371	71	88	299	8.2
160	58		28	Normal				1	1	2	186	79	17	864	9.8
161	57		83	Normal				174	0	49	429	74	19	1671	10.7
162	57		28	Normal		INS	Allo-SCT	18	1	64	375	64	245	98	6.6

163	64	yes	77	42-45, XY		INS		169	0	65	495	206	150	680	9.4
164	62		60	Normal				2	1	127	146	41	355	17	4.1
166	55		25	Normal			Allo-SCT	2	1	15	329	70	61	378	8.6
167	68		50	Normal				25	1	4	296	67	110	180	7.5
169	55	yes	86	46, XX	ITD	INS	Allo-SCT	5	1	32	351	82	106	272	8.1
170	58		60	+8	ITD			0	1	54	612	98	20	3018	11.6
172	48	yes	76	Normal	ITD	INS		7	1	234	527	86	118	384	8.6
173	26		70	Normal		INS		162	0	8	486	77	60	623	9.3
175	23			Normal			Allo-SCT	174	0	103	1157	56	113	573	9.2
176	69		87	45-47, X, -X, -17, +3				110	1	49	607	65	172	229	7.8
177	70	yes	74	Normal	ITD			1	1	57	1273	79	35	2873	11.5
180	66		21					19	1	2	187	78	47	310	8.3
181	64		90	Complex				13	1	30	279	82	25	915	9.8
183	69	yes	77			INS		2	1	294	2428	56	28	4856	12.2
184	45		90	Normal		INS	Allo-SCT	39	1	54	536	63	113	299	8.2
187	20		81	Normal	ITD		Allo-SCT	27	1	24	1636	81	63	2103	11.0
188	42		85	Normal	ITD	INS		8	1	61	570	84	179	267	8.1
189	50		30	-7				2	1	50	196	54	17	623	9.3
191	69		90	t(16;16)	ITD			18	1	25	275	91	101	248	8.0
192	66		97	Normal	ITD	INS		5	1	109	662	129	29	2899	11.5
195	65		21	Normal				1	1	28	179	44	19	415	8.7
197	47			+8			Allo-SCT	5	1	16	380	58	16	1378	10.4
198	42		77	Normal	ITD		Allo-SCT	133	0	66	1879	80	70	2147	11.1
199	60		34	Normal				3	1	<1	171	66	139	81	6.3
201	68		27	Normal				1	1	33	323	66	15	1421	10.5
203	67		35	Complex				0	1	6	160	72	14	823	9.7
204	54		50	Normal	ITD	INS		7	1	32	561	47	170	155	7.3
206	66	yes	93	47-48, XX, +mar,inc				15	1	10	191	96	190	97	6.6
207	62	yes	15	+8, +21	ITD		Allo-SCT	9	1	61	198	70	19	729	9.5
208	64		25	Normal			Allo-SCT	9	1	3	205	79	81	200	7.6

209	59		55	Normal	ITD			6	1	8	591	94	632	88	6.5
212	63	yes	68	Normal		INS		2	1	37	746	106	31	2551	11.3
217	54		52	Normal	ITD			5	1	3	536	157	9	9350	13.2
218	62		54	-7			Allo-SCT	14	1	21	236	62	97	151	7.2
220	37					INS		107	0	4	168	63	220	48	5.6

TABLE S2 – EASIX and mEASIX scores for individual patients: a presentation of the calculated and the log2 transformed pretreatment scores.

The table presents the patient id number together with the EASIX and mEASIX scores for each of the 160 patients included in the present study.

Patient	EASIX	Log2(EASIX)	mEASIX	Log2(mEASIX)
1	143	7.2	29	4.9
2	38	5.2	2	1.2
3	237	7.9	93	6.5
4	95	6.6	253	8.0
5	7977	13.0	295	8.2
6	1895	10.9	217	7.8
7	773	9.6	1584	10.6
8	78	6.3	37	5.2
9	546	9.1	48	5.6
10	639	9.3	3982	12.0
11	3086	11.6	3051	11.6
12	340	8.4	135	7.1
13	262	8.0	3	1.6
14	1924	10.9	2958	11.5
15	1243	10.3	1940	10.9
16	33	5.0	0	-1.1
17	162	7.3	433	8.8
18	696	9.4	22	4.4
19	232	7.9	476	8.9
20	207	7.7	3	1.6
21	584	9.2	37	5.2
22	317	8.3	25	4.6
23	683	9.4	273	8.1

24	3607	11.8	190	7.6
25	2564	11.3	5636	12.5
26	162	7.3	30	4.9
27	307	8.3	640	9.3
28	107	6.7	3	1.6
29	331	8.4	41	5.3
30	2333	11.2	4704	12.2
31	1741	10.8	114	6.8
32	665	9.4	242	7.9
33	597	9.2	1078	10.1
34	9713	13.2	413	8.7
35	781	9.6	192	7.6
36	102	6.7	6	2.6
37	801	9.6	736	9.5
38	3058	11.6	5345	12.4
39	1406	10.5	755	9.6
40	483	8.9	148	7.2
41	4901	12.3	496	9.0
42	238	7.9	78	6.3
43	248	8.0	347	8.4
44	179	7.5	146	7.2
45	2162	11.1	5037	12.3
46	68	6.1	83	6.4
47	173	7.4	31	5.0
48	1795	10.8	3519	11.8
49	853	9.7	1797	10.8
50	242	7.9	49	5.6
51	961	9.9	139	7.1
52	1314	10.4	472	8.9
53	708	9.5	46	5.5

54	243	7.9	87	6.4
55	145	7.2	172	7.4
56	3654	11.8	2266	11.1
57	2378	11.2	2254	11.1
58	78	6.3	4	1.8
59	102	6.7	18	4.2
60	79	6.3	3	1.5
61	269	8.1	720	9.5
62	200	7.6	1221	10.3
63	267	8.1	489	8.9
64	69	6.1	4	1.9
65	307	8.3	282	8.1
66	1871	10.9	63	6.0
67	1399	10.5	657	9.4
68	1017	10.0	952	9.9
69	2853	11.5	7224	12.8
70	789	9.6	112	6.8
71	1046	10.0	112	6.8
72	902	9.8	267	8.1
73	263	8.0	158	7.3
74	3333	11.7	51	5.7
75	82	6.4	4	2.1
76	357	8.5	50	5.6
77	3730	11.9	3637	11.8
78	316	8.3	19	4.3
79	310	8.3	184	7.5
80	7076	12.8	3004	11.6
81	508	9.0	122	6.9
82	869	9.8	1037	10.0
83	680	9.4	80	6.3

84	1925	10.9	2688	11.4
85	74	6.2	3	1.4
86	258	8.0	94	6.6
87	183	7.5	490	8.9
88	201	7.7	287	8.2
89	371	8.5	1577	10.6
90	1764	10.8	169	7.4
91	794	9.6	112	6.8
92	19	4.2	259	8.0
93	1097	10.1	113	6.8
94	303	8.2	339	8.4
95	7832	12.9	3517	11.8
96	3067	11.6	6930	12.8
97	1501	10.6	1372	10.4
98	332	8.4	17	4.1
99	80	6.3	1	0.1
100	154	7.3	707	9.5
101	64	6.0	26	4.7
102	57	5.8	1	-0.2
103	11061	13.4	42519	15.4
104	606	9.2	312	8.3
105	117	6.9	3	1.6
106	368	8.5	11	3.4
107	2932	11.5	3657	11.8
108	1405	10.5	755	9.6
109	547	9.1	105	6.7
110	805	9.7	952	9.9
111	223	7.8	298	8.2
112	353	8.5	71	6.1
113	5637	12.5	1847	10.9

114	387	8.6	52	5.7
115	145	7.2	172	7.4
116	1859	10.9	20	4.3
117	139	7.1	87	6.4
118	1764	10.8	169	7.4
119	2661	11.4	2461	11.3
120	234	7.9	35	5.1
121	316	8.3	221	7.8
122	299	8.2	38	5.2
123	864	9.8	22	4.5
124	1671	10.7	1106	10.1
125	98	6.6	98	6.6
126	680	9.4	215	7.7
127	17	4.1	52	5.7
128	378	8.6	81	6.3
129	180	7.5	11	3.4
130	272	8.1	106	6.7
131	3018	11.6	1652	10.7
132	384	8.6	1045	10.0
133	623	9.3	65	6.0
134	573	9.2	1055	10.0
135	229	7.8	173	7.4
136	2873	11.5	2073	11.0
137	310	8.3	8	3.0
138	915	9.8	335	8.4
139	4856	12.2	25494	14.6
140	299	8.2	256	8.0
141	2103	11.0	623	9.3
142	267	8.1	194	7.6
143	623	9.3	576	9.2

144	248	8.0	68	6.1
145	2899	11.5	2488	11.3
146	415	8.7	264	8.0
147	1378	10.4	380	8.6
148	2147	11.1	1772	10.8
149	81	6.3	1	0.3
150	1421	10.5	711	9.5
151	823	9.7	69	6.1
152	155	7.3	106	6.7
153	97	6.6	10	3.3
154	729	9.5	636	9.3
155	200	7.6	8	2.9
156	88	6.5	7	2.9
157	2551	11.3	890	9.8
158	9350	13.2	179	7.5
159	151	7.2	51	5.7
160	48	5.6	3	1.6
